# Rehabilitation of Upper Extremity Nerve Injuries Using Surface EMG Biofeedback: Protocols for Clinical Application

**DOI:** 10.3389/fnins.2018.00906

**Published:** 2018-12-04

**Authors:** Agnes Sturma, Laura A. Hruby, Cosima Prahm, Johannes A. Mayer, Oskar C. Aszmann

**Affiliations:** ^1^Christian Doppler Laboratory for Restoration of Extremity Function, Department of Surgery, Medical University of Vienna, Vienna, Austria; ^2^Health Assisting Engineering, University of Applied Sciences FH Campus Wien, Vienna, Austria; ^3^Neuromechanics and Rehabilitation Technology Group, Department of Bioengineering, Imperial College London, London, United Kingdom; ^4^Division of Plastic and Reconstructive Surgery, Department of Surgery, Medical University of Vienna, Vienna, Austria

**Keywords:** nerve reconstruction, upper extremity rehabilitation, surface electromyography, neuro-rehabilitation, nerve transfer, prosthetic rehabilitation

## Abstract

Motor recovery following nerve transfer surgery depends on the successful re-innervation of the new target muscle by regenerating axons. Cortical plasticity and motor relearning also play a major role during functional recovery. Successful neuromuscular rehabilitation requires detailed afferent feedback. Surface electromyographic (sEMG) biofeedback has been widely used in the rehabilitation of stroke, however, has not been described for the rehabilitation of peripheral nerve injuries. The aim of this paper was to present structured rehabilitation protocols in two different patient groups with upper extremity nerve injuries using sEMG biofeedback. The principles of sEMG biofeedback were explained and its application in a rehabilitation setting was described. Patient group 1 included nerve injury patients who received nerve transfers to restore biological upper limb function (*n* = 5) while group 2 comprised patients where biological reconstruction was deemed impossible and hand function was restored by prosthetic hand replacement, a concept today known as bionic reconstruction (*n* = 6). The rehabilitation protocol for group 1 included guided sEMG training to facilitate initial movements, to increase awareness of the new target muscle, and later, to facilitate separation of muscular activities. In patient group 2 sEMG biofeedback helped identify EMG activity in biologically “functionless” limbs and improved separation of EMG signals upon training. Later, these sEMG signals translated into prosthetic function. Feasibility of the rehabilitation protocols for the two different patient populations was illustrated. Functional outcome measures were assessed with standardized upper extremity outcome measures [British Medical Research Council (BMRC) scale for group 1 and Action Research Arm Test (ARAT) for group 2] showing significant improvements in motor function after sEMG training. Before actual movements were possible, sEMG biofeedback could be used. Patients reported that this visualization of muscle activity helped them to stay motivated during rehabilitation and facilitated their understanding of the re-innervation process. sEMG biofeedback may help in the cognitively demanding process of establishing new motor patterns. After standard nerve transfers individually tailored sEMG biofeedback can facilitate early sensorimotor re-education by providing visual cues at a stage when muscle activation cannot be detected otherwise.

## Introduction

**FIGURE 1:**
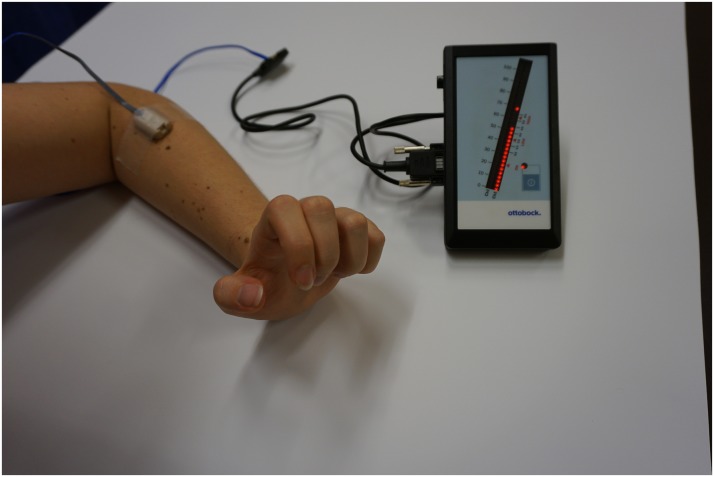
Training with the MyoBoy (Ottobock, Duderstadt, Germany) with one dry electrode placed on the extensor compartment of the forearm. The EMG signal’s amplitude is reflected by the LED dots. This set-up may be used for home training.

**FIGURE 2:**
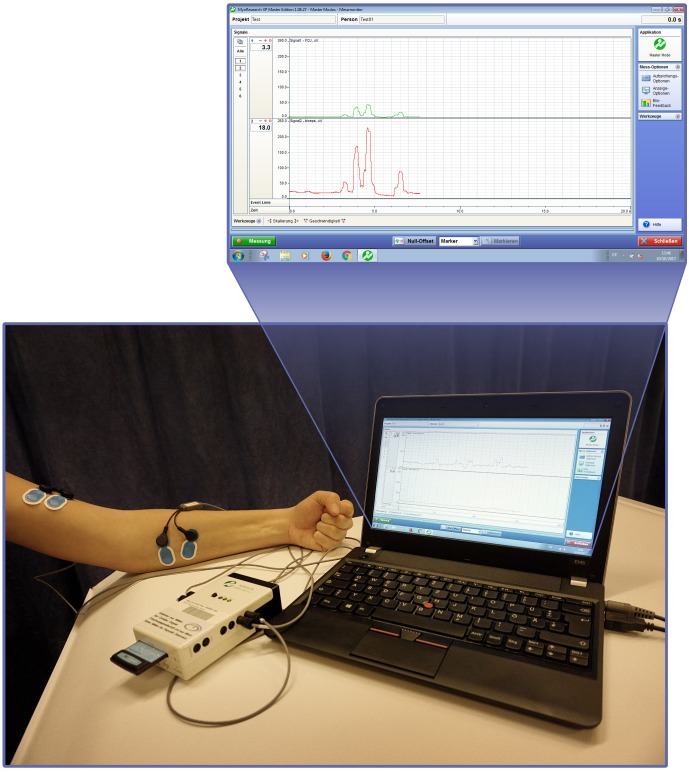
Surface EMG biofeedback set-up with the TeleMyo system (Noraxon, United States) and screenshot of the TeleMyo-Software simultaneously recording two EMG signals, represented by color-coded graphs.

Biofeedback applications measure biological information and feed them back to the patient to increase awareness and control over biological processes (Neblett, 2016). With the advent of information technology, computerized multimedia displays allow highly sophisticated and detailed recordings of real-time biological data that otherwise would not be identified by both patient and clinician (Giggins et al., 2013). Representing one of the oldest biofeedback modalities sEMG provides feedback of muscle activity by conversion of myoelectrical activity into visual and/or auditory information (Cram, 2003; Giggins et al., 2013; Kim, 2017), e.g., displayed as color-coded graphs on a computer screen with the device itself in front of the patient, as shown in Figures 1, 2. While Figure 1 shows training with a stand-alone 2-channel device with dry electrodes (MyoBoy^®^ by Ottobock Healthcare, Duderstadt, Germany), Figure 2 includes a set-up with wet electrodes and device software used to display muscular activity (TeleMyo 2400T G2^®^ by Noraxon, United States). As illustrated in these figures wet electrodes have a thin coating of conductive gel on their surface, which supports electrical conductivity and makes them self-adhesive, but also allows single-use only. In contrast to that dry electrodes do not use any gel and need to be attached to the skin (e.g., with tape).

Nerve injuries of the upper extremity may cause substantial loss of motor and sensory function resulting in alterations in both the peripheral and central nervous system (CNS) which may continue through recovery (Novak and Von Der Heyde, 2013, 2015). Today, nerve transfer surgery plays a major role in nerve reconstruction, particularly in severe proximal nerve injuries (Tung and Mackinnon, 2010). Upon nerve transfer surgery (neurotization) an intact motor nerve from one muscle (donor nerve) is redirected to the distal undamaged portion of a nerve from another muscle (recipient nerve), effectively bypassing the injured segment of the nerve (Liu et al., 2012). Following nerve injury, timely reconstruction should be initiated since degeneration and fibrosis of motor end plates occurring within 1–2 years may preclude successful muscle re-innervation (Terzis and Papakonstantinou, 2000). Furthermore, in upper limb amputees, the concept of selective nerve transfers, known as targeted muscle re-innervation (TMR), has dramatically improved prosthetic arm and hand function (Kuiken et al., 2004, 2007; Dumanian et al., 2009).

It is well known that damage to peripheral nerves inevitably creates change at a central level, i.e., cortical reorganization which occurs following deafferentation of a respective area (Pons et al., 1991; Elbert et al., 1994; Flor, 2008). With increasing performance of nerve transfers and expanded clinical experience, experts in the field of nerve reconstruction have come to appreciate the important role of cortical plasticity and motor relearning during functional recovery following a nerve transfer (Anastakis et al., 2008). It has been shown that recovery after surgical nerve reconstruction is both a function of peripheral nerve regeneration and adaptations within the CNS, making use of the brain’s plastic capacity (Dahlin et al., 2017).

As an example, intercostal-to-musculocutaneous nerve transfers are commonly used to re-innervate the biceps muscle in global brachial plexopathies (Millesi, 1977; Narakas, 1978; Terzis and Kostopoulos, 2007; Xiao et al., 2014). Upon successful regeneration of axons, motor control of the re-innervated biceps muscle initially requires activation of the intercostal nerves, i.e., through breathing and/or coughing (Carlstedt et al., 2004). Cognitive rehabilitation capitalizing on CNS plasticity allows patients to re-educate their brain and to gain volitional control of elbow flexion without activation of former intercostal nerve territories (Dahlin et al., 2017). Functional magnetic resonance imaging studies have shown that in patients with good biceps muscle re-innervation, induced and localized activity in the former biceps muscle cortical area is re-established, indicating cortical plasticity following successful nerve reconstruction (Malessy et al., 2003). Therefore, the importance of cortical changes and plasticity need not be underestimated during rehabilitation following motor nerve transfers (Novak, 2008).

Sensorimotor re-education following complex nerve reconstruction is a cognitively demanding process necessitating a structured neuro-rehabilitation program (Novak, 2008; Bergmeister et al., 2017). sEMG biofeedback has been widely used for rehabilitation of the upper extremity in stroke patients (Rayegani et al., 2014; Kim, 2017). In the nerve transfer patient, however, this biofeedback technique has not yet been described. Following nerve transfer surgery, the regeneration of motor axons requires a considerable period of time and patients will often struggle to attain control of volitional contractions in the re-innervated muscle (Kahn and Moore, 2016). Before visual or even palpable contractions occur sEMG can provide valuable feedback for the patient and guide rehabilitation focused on sensorimotor re-education. With the establishment of new motor patterns and cortical remapping, control of the re-innervated muscle will be attained without activation of the donor muscle after successful rehabilitation (Novak and Von Der Heyde, 2013).

Here, we introduce two rehabilitation protocols using surface EMG-guided biofeedback in different groups of nerve injury patients. The first group of patients includes patients with severe nerve injuries of the upper extremity undergoing nerve transfers to restore biological arm and hand function. The second group includes patients in whom biological reconstruction has failed and extremity function was reconstructed with a myoelectric prosthesis.

## Protocols

### Rehabilitation Protocol Using Surface EMG Biofeedback for Patients With Nerve Transfers to Restore Biological Upper Extremity Function

**FIGURE 3:**
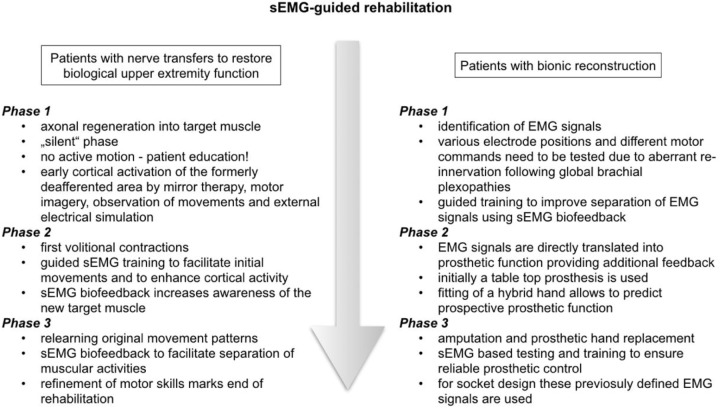
Rehabilitation process of both patient groups.

**FIGURE 4:**
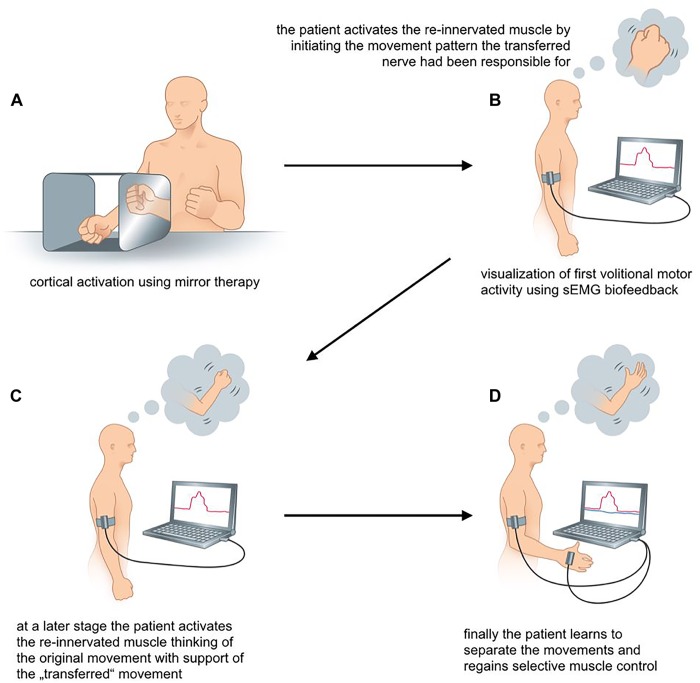
**(A–D)** sEMG-guided rehabilitation for patients with biologic reconstruction of upper extremity function. The scheme illustrates the rehabilitation process following an Oberlin’s ulnar nerve transfer.

Rehabilitation after nerve transfers is divided into three phases. In the *first phase* following surgery the nerves regenerate and no active motion is possible, referred to as “silent” phase (see Figure 3). This re-innervation process usually takes a considerable period of time. Therapy in this early stage, however, can be initiated for cortical activation by mirror therapy (see Figure 4A), motor imagery and observation of movements (McCabe, 2011; Bowering et al., 2012). In mirror therapy, originally described to treat phantom limb pain by Ramachandran and Hirstein (1998), a patient places his normal hand on one side of a vertically placed mirror, which creates the illusion that the injured, amputated or denervated hand has returned and exhibits normal function. External electrical muscle stimulation may be of use to elicit movement of the paralyzed limb area, which also enhances cortical activation. This approach supports motor learning at a later stage. Additionally, therapy might also focus on body symmetry, trunk stability, and posture as well as preservation of range of motion for joints of the affected extremity.

The first volitional activation of the re-innervated muscle marks the start of the *second phase* of rehabilitation. Between 3 and 6 months post surgery we recommend monthly assessments of muscle activity using transcutaneous electrodes to identify first volitional muscle activation. The initial re-innervation is confirmed, when the sEMG signal of the muscle activation repeatedly has an amplitude that is 2–3 times higher than the amplitude during relaxation. This allows patient and therapist to see a distinct difference between muscle relaxation and activation. sEMG biofeedback training increases awareness of the new target muscle. Firstly, patients may not know how to activate a new target muscle. This is because activation requires initiation of movement patterns that the nerve had before its transfer (Novak and Von Der Heyde, 2015). For example, in case of Oberlin’s ulnar nerve transfer, where a fascicular group of the ulnar nerve is transferred to the musculocutaneus nerve (Oberlin et al., 1994), the patient initially activates the biceps by thinking about “hand closing” or “activating the flexor carpi ulnaris (FCU)” (Oberlin et al., 2002). As this may be contra-intuitive for the patient without profound knowledge of the underlying anatomy, perioperative patient education is crucial. It ensures that they understand the consequence of nerve injury, the surgical procedure of the nerve transfer and the expected recovery (Novak and Von Der Heyde, 2013; Kahn and Moore, 2016).

By using sEMG biofeedback the therapist can identify individual, suitable movements for reliable muscular activation as an electrode is placed over the muscle of interest and the patient is asked to perform specific movements that the transferred nerve is originally responsible for (see Figure 4B). Additionally, sEMG is used to visualize muscle contraction during training, which is not visible or even palpable at that early stage of re-innervation. As soon as the patient knows how to activate the re-innervated muscle, he might think of a combination of the original muscle movement and the new activation pattern. In case of an Oberlin’s ulnar nerve transfer this might include “elbow flexion” in combination with “hand closing” (see Figure 4C). As suggested by Novak, bilateral actions, i.e., performing the movement with both the injured and the healthy side, can be helpful for some patients (Novak, 2008; Novak and Von Der Heyde, 2013).

The *third phase* of rehabilitation starts as soon as the muscle strength is sufficient to overcome the inertia of the corresponding joint and initiate actual movement. Here, the focus lies on relearning the original movement pattern. After an Oberlin’s nerve transfer this means flexion of the elbow without simultaneously closing the hand (see Figure 4D). The therapist encourages the patient to gradually activate the re-innervated muscle with decreasing activity of the supporting movements. Additionally, closing the hand without simultaneous contraction of the biceps muscle needs to be promoted through training. To support this cognitively demanding process, we encourage the use of sEMG biofeedback to attain reliable separation of muscle activity. Here, a setup with two EMG channels is recommended. One electrode is placed on the re-innervated muscle and the other on the original donor nerve muscle. This simultaneously visualizes the activity of both muscles. As shown in Figure 4D activation of one muscle without the other can be trained. The direct feedback using sEMG recordings provides the therapist as well as the patient with precise information about desirable and undesirable strategies for motor task execution. By the end of this third phase muscle force and fine motor skills should ideally meet the patient’s as well as the clinician’s expectations.

### Rehabilitation Protocol Using Surface EMG Biofeedback for Patients With Bionic Reconstruction

The primary rehabilitation goal for patients eligible for bionic reconstruction is not to recover muscle strength. Instead, rehabilitation aims at establishing two independent EMG signals needed for reliable control of a myoelectric prosthesis after elective amputation (Salminger et al., 2016). The surgical concept and detailed treatment algorithm for bionic reconstruction can be found elsewhere (Aszmann et al., 2015; Hruby et al., 2017). In most global BP patients residual myoactivity may be detected in the fore- and upper arm, which – although without clinical significance – suffices to control a prosthetic hand. In these patients, sEMG training can be initiated without delay. In others, nerve and/or muscle transfers are needed to create additional EMG signals for future prosthetic control. sEMG training in these cases, therefore, starts with first volitional contractions of the new target muscles, approximately 6–9 months after nerve and/or muscle transfer. In this group, regular follow-ups where sEMG activity is assessed and documented, take place at 3, 6, and 9 months after surgery. In our experience nerve regeneration takes longer in this patient group and first volitional muscle activation is seldom detected before 6 months after surgery. As for patients with biological reconstruction of function, the initial re-innervation is confirmed, when the sEMG signal of the muscle activation repeatedly has an amplitude that is 2–3 time higher than the amplitude during relaxation.

The *first phase* of training includes the identification of EMG signals. The definition of the best positions for recording sEMG is critical and many possible electrode positions need to be compared by observing the amplitude of the EMG signal. Due to the aberrant re-innervation of muscles after global brachial plexopathies, the identification of movements that result in the greatest muscle activity, is usually complex. Cognitive motor commands might elicit movements which differ from biological patterns, e.g., the signal for closing the hand may be located at the dorsal aspect of the fore-arm. Therefore, even movements that seem illogical, or rather anatomically incorrect need to be tested. The advantage of sEMG in contrast to needle EMG arises from the possibility to adjust the electrode’s position multiple times during testing, which is not feasible with needle EMG and might also cause pain. Additionally, the identification of sEMG signals is more relevant as later on transcutaneous electrodes within the prosthetic socket pick up these EMG signals and translate them into prosthetic hand function.

**FIGURE 5:**
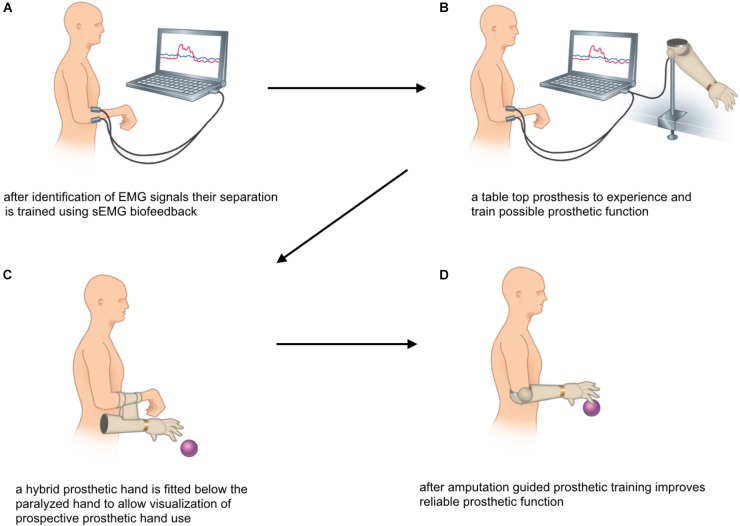
**(A–D)** sEMG-guided rehabilitation for patients with bionic hand reconstruction.

As soon as two different electrode locations with their unique activation pattern are established, sEMG training focuses on the separation of these signals (see Figure 5A). With the muscular activity visualized by the sEMG feedback device the patient tries to activate one muscle without the other. Also here, the direct feedback allows patient and therapist to try slight variations of the movements to find the best starting point for selective and consistent muscular activity.

During the *second phase* of rehabilitation the sEMG signal can be used for direct control of a table top prosthesis (as shown in Figure 5B). Although the prosthetic hand does not give as precise feedback as the visualization via EMG graphs, this approach allows to predict prosthetic hand function after amputation. Finally, patients are fitted with a hybrid hand, a fully functional prosthesis mounted on to or below the paralyzed hand. This gives a more realistic outlook on future prosthetic hand function, as illustrated in Figure 5C.

Elective amputation marks the beginning of the *third phase* of rehabilitation. sEMG based testing and training ensures that the patient can still activate the two muscles independently. The signal positions can also be used for the design of the prosthetic socket, which is usually possible 4–6 weeks after amputation. As recommended in all amputees (Johnson and Mansfield, 2014; Resnik et al., 2014) regular prosthetic training (see Figure 5D) optimizes device control in activities of daily living and marks the end of rehabilitation.

## Patients, Methods, and Design of Feasibility Study

We implemented the described protocols into clinics in eleven patients to test whether their application was feasible and help improve outcomes.

### Patients

Patients who followed the described protocols had a severe injury of one or several peripheral nerves of the upper extremity that required a surgical reconstruction. Exclusion criteria were injuries of the CNS, untreated psychological disorders and unstable fractures of the upper extremity.

Depending on the injury and the intervention planned, patients were treated with either one of the rehabilitation protocols:

• Group 1: Patients with peripheral nerve injuries and selective nerve transfers to reconstruct biological upper limb function (*n* = 5).
• Group 2: Patients with severe peripheral nerve injuries, where biological reconstruction was deemed impossible. In these patients, prosthetic devices were used to restore hand function by technological means (*n* = 6). The concept of bionic reconstruction was recently described by Aszmann et al. (2015) and Hruby et al. (2017).

Patient characteristics can be found in Tables 1, 2. All patients received structured training with sEMG biofeedback. A summary of both rehabilitation approaches can be found in Figure 3.

**Table 1:** Patient characteristics of Group 1, in whom biological restoration of upper limb function was performed.

Case nr.	Sex, age (years)	Type of accident	Type of lesion	Reconstructive surgeries for restoration of upper limb function
1	m, 68	Motorcycle accident	Polytrauma; global brachial plexopathy	Nerve grafts to bridge defect of MCN; thoracodorsal nerve grafts to bridge defect of axillary nerve; nerve grafts for posterior trunk reconstruction; Oberlin’s ulnar nerve transfer to MCN motor branch to the short head of the biceps
2	m, 56	Bicycle accident	Nerve root avulsion of C5-C6	Oberlin’s ulnar nerve transfer to MCN motor branch for restoration of biceps function; transfer of radial triceps motor branch to axillary nerve
3	m, 62	Bicycle accident	Extensive damage to superior trunk of the BP; traction injury of C7	XI-to-suprascapular nerve transfer; end-to-end transfer of phrenic nerve to C7; transfer of ulnar nerve fascicle to biceps motor branch of MCN; transfer of median nerve fascicle to brachialis motor branch of MCN; transfer of radial nerve fascicle to axillary nerve
4	f, 22	Car accident	Nerve root avulsion of C7; damage to C8 and T1	Nerve grafts from C5 and C6 to MCN, median and radial nerve; nerve grafts from C8 to median, radial and ulnar nerve; nerve grafts from T1 to ulnar nerve
5	f, 43	Minor trauma years after OBPL	Traction injury of superior and medial trunk of the BP	Nerve grafts to bridge defect of C5, C6, and C7 to restore elbow function and shoulder stability; transfer of median nerve fascicle to brachial motor branch of MCN


*BP, brachial plexus; MCN, musculocutaneous nerve; OBPL, obstetrical brachial plexus lesion; OP, operation; XI, spinal accessory nerve.*

**Table 2:** Patient characteristics of Group 2, in whom bionic reconstruction was initiated due to infeasibility of biological treatment alternatives.

Case nr.	Sex, age (years)	Type of accident	Type of lesion	Surgeries to improve biotechnological interface after initial reconstructions have failed to improve hand function
1	m, 32	Fall from height	Avulsion of C7–T1, traction injury of the infraclavicular plexus	Elective amputation of the forearm
2	m, 32	Motorcycle accident	Rupture of all 3 trunci of the BP	Free gracilis muscle transferred to forearm extensor compartment & neurotization of deep branch of radial nerve to obturator nerve; elective amputation of the forearm
3	m, 55	Motorcycle accident	Avulsion of C5-T1	Elective amputation of the upper arm
4	m, 38	Motorcycle accident	Extensive damage to roots C5-C8; avulsion of T1	Elective amputation of the forearm
5	m, 27	Motorcycle accident	Avulsion C8-T1	Elective amputation of the forearm
6	m, 43	Motorcycle accident	Avulsion of C6-T1	Transfer of triceps muscle to supraspinatus fossa and transfer of biceps muscle to supraclavicular fossa to improve prosthetic fitting; elective amputation of the arm (shoulder exarticulation)


*Surgeries may include selective nerve and muscle transfers to establish myoactivity in the fore- and upper arm, which will then drive a myoelectric prosthetic hand. Elective amputation is either performed at a transradial or transhumeral level, depending on the residual muscle activity. All selective nerve transfers performed in this patient group were successful.*

This clinical implementation was approved by the ethics committee of the Medical University of Vienna, Austria and carried out in accordance with the standards set by the Declaration of Helsinki. All patients provided written informed consent to participating in this study.

### Materials

The EMG electrodes used in this study were bipolar and included a ground, circumventing the need of an extra ground electrode [product number: 13E202 = 50 (50 Hz), Ottobock Healthcare, Duderstadt, Germany].

All patients in group 2 used a SensorHand Speed© (Ottobock Healthcare, Duderstadt, Germany) as their standard prosthetic device.

### Implementation of Rehabilitation Protocols

In both groups the suggested procedures of the protocols could be implemented for all patients by one experienced therapist (AS). In group 1 differences in between subjects included the use of external electrical stimulation after nerve transfer surgery (*n* = 3 users, Cases 2, 3, and 4; *n* = 2 non-users, Cases 1 and 5) using exponential current for denervated muscles and surge current for previously re-innervated muscles. In group 2 the myosignal identification for future prosthetic control was complicated by the mixed pattern of BP injury and the aberrant re-innervation that had occurred. Through the application of sEMG feedback, however, signals could be readily detected with multiple electrode positions and various motor commands guided by the therapist. The process of sEMG signal identification therefore lasted several hours as various, oftentimes counter-intuitive motor commands needed to be tested in order to elicit contraction in the target muscle. For example, in Case 2 of group 2 aberrant re-innervation caused the signal for closing the hand to be located at the dorsal aspect of the fore-arm. Although the location of sEMG signals and corresponding motor commands to elicit contraction greatly varied inter-individually, we found that the majority of signals were located at the proximal third of the fore-arm (mostly pronator teres muscle, and extensor compartment). The time between nerve transfer surgery and first volitional muscle activation is outlined in Tables 3, 4.

**Table 3:** Upper limb function of patients with biologic reconstruction of hand function (patient group 1) before treatment and after end of therapy.

Case nr.	Upper limb function including BMRC grades at baseline	Upper limb function including BMRC grades at follow-up	Time between nerve transfer surgery and first volitional sEMG activity	No. of therapy sessions in total (30 min each)
1	Deltoid muscle: 0	Deltoid muscle: 2	5 months	25
	Elbow flexion: 0	Elbow flexion: 3		
	Triceps muscle: 0	Triceps muscle: 2		
	No active hand	Wrist extension: 1		
	function	Finger extension: 2		
2	Elbow flexion: 1	Elbow flexion: 5	4 months	22
	Deltoid muscle: 2-	Deltoid muscle: 5		
3	Elbow flexion: 0	Elbow flexion: 5	3 months	30
	Deltoid muscle: 0	Deltoid muscle: 4		
	Triceps muscle: 3	Triceps muscle: 5		
	Wrist extension: 3+	Wrist extension: 5		
	Finger flexion: 3+	Finger flexion: 5		
4	Elbow flexion: 0	Elbow flexion: 3+	5 months	20
	Triceps muscle: 0	Triceps muscle: 2		
	No active hand function	Wrist flexion: 3 Finger flexion (ulnar FDP part): 3		
5	Elbow flexion: 0	Elbow flexion: 3	4 months	18
	Deltoid muscle: 2	Deltoid muscle: 2		
	Triceps muscle: 3+	Triceps muscle: 4		
**Mean (±SD)**			4.2 ± 0.75 months	23 ± 4.20


*The assessment of muscle strength using the British Medical Research Council (BMRC) scale was based on the pre-operative type of lesion, the reconstructive surgery performed and the associated motor function to be recovered. There was no functional impairment of the muscles not included in the table. In all patients shoulder and elbow function was impaired at baseline and improved to follow-up. Additionally, the time between surgery and start of sEMG training and the number of therapy sessions for each patient are presented.*

**Table 4:** Scores of patients with bionic reconstruction (patient group 2) before treatment and after final prosthetic fitting.

Case nr.	ARAT at baseline	ARAT at follow-up	Start of sEMG training	No. of therapy sessions in total (30 min each)
1	7	35	Immediately after first consultation	24
2	0	15	Training with one signal immediately after first consultation; second signal was available 9 months after free gracilis muscle transfer + nerve transfer	30
3	0	19	Immediately after first consultation	16
4	1	22	Immediately after first consultation	20
5	9	42	Immediately after decision to aim for a bionic reconstruction as biologic reconstruction failed	20
6	0	17	Immediately after first consultation	22
**Mean (±SD)**	2.83 ± 4.07	25.00 ± 10.94		22 ± 4.32


*In the Action Research Arm Test (ARAT), a maximum of 57 points is attainable representing normal hand function. As indicated in the table, patients had hardly any function in their upper extremity at baseline (mean 2.83), but regained some useful function after bionic reconstruction (mean 25.00), which was statistically significant. Additionally, the starting point for sEMG training and the number of therapy sessions for each patient are presented.*

In both groups time in therapy depended on the patients’ time limitations and the extent of injury. Individual adaptations were made with each patient. Guided training sessions using sEMG biofeedback with a therapist lasted 30 min to preclude muscle fatigue, which were usually offered once every 2 weeks for group 2. In group 1 time in therapy was intensified to once per week during phase 2 to support motor re-education, whereas during phases 1 and 3 patients received therapy once a month.

The number of therapy sessions for each individual patient can be found in Tables 3, 4. All patients had the possibility to use EMG home training devices (see Figure 1), which was accepted by nine of eleven patients. The patients using the home training device all reported to have used it regularly and said it increased their training motivation due to intuitive feedback on muscle activity.

### Design of the Feasibility Study

This was a within subjects pre- and post-test study. The baseline measurements of participants’ upper limb function were performed after peripheral nerve injury and prior to surgical and therapeutical intervention. The follow-up measurements were conducted after the patients were discharged from rehabilitation.

### Functional Outcome Measures

To evaluate hand and arm function, the British Medical Research Council (BMRC) (James, 2007) was used to assess muscle strength in patients with biological reconstruction (group 1). This grading system is the standard measure of muscle function after peripheral nerve injuries (Prosser and Conolly, 2005). In group 2 (bionic reconstruction) the ARAT (Action Research Arm test) was used to assess upper limb function (Lyle, 1981). This observational test consists of four sections with different tasks and a score maximum of 57 points (Lyle, 1981). It was performed before amputation (with the functionless “plexus” hand) as well as after final prosthetic fitting with the prosthetic hand.

### Statistics

In accordance with the limited sample size of this study, in group 2 non-parametric tests were performed for the ARAT scores as these did not meet the requirement for normal distributions. Therefore, a paired 2-tailed Mann–Whitney *U*-test was used for the analysis. The significance level was set at Cronbach alpha = 0.05. Explorative statistics were applied in group 1 for the BMRC grades. Statistical analysis was performed in SPSS 24 (IBM, Armonk, NY, United States).

## Results

Functional outcome measures for group 1 (biological reconstruction of upper limb function) are outlined in Table 3. Table 4 displays functional outcome measures for group 2 (bionic reconstruction with prosthetic hand replacement). All cases showed an improvement of hand function at the follow-up. The mean ARAT score improved significantly from 2.83 ± 4.07 to 25.00 ± 10.94 (*p* = 0.028). In group 1, shoulder and elbow function could be improved in all patients as measured by the BMRC scale. All patients regained an active elbow flexion against gravity (with scores obtained between M3 and M5).

## Discussion

After peripheral nerve injury, immediate changes in the peripheral but also in the CNS occur, which continue through re-innervation and recovery (Novak and Von Der Heyde, 2015). Practice, repetition, and structured training programs with appropriate biofeedback are necessary to establish correct motor patterns (Novak and Von Der Heyde, 2013). Biofeedback using sEMG recordings has been shown to facilitate significant clinical improvements and to enhance the rehabilitation process in various neuromuscular diseases such as in stroke (Giggins et al., 2013; Huang et al., 2013; Oravitan and Avram, 2013; Neblett, 2016). In this paper we presented a structured rehabilitation protocol using sEMG biofeedback in patients with severe nerve injuries. Our clinical application included patients receiving nerve transfers to restore biological upper limb function as well as patients who underwent nerve surgeries to improve the future biotechnological interface, elective amputation and prosthetic hand replacement.

During the past decades, the use of nerve transfers has expanded with a wider range of applications and improved functional outcomes, particularly to restore biological extremity function in patients with severe proximal nerve injuries (Bertelli and Ghizoni, 2004; Novak, 2008; Bertelli and Ghizoni, 2010; Tung and Mackinnon, 2010; Mackinnon et al., 2012; Mackinnon, 2016). Still, waiting for a muscle function to recover is one of the greatest challenges for a patient after undergoing nerve transfer surgery. Especially in the early post-operative phase patients may be frustrated and/or depressed when no motor activity is seen (Kahn and Moore, 2016). This time period, where the patient feels that “nothing happens,” is possibly shortened with the use of sEMG feedback as faint muscle activity is visualized before it is visible or even palpable. sEMG set-ups are valuable tools to localize those parts of a muscle with weak contractile actions, which would otherwise be unnoticed to the patient allowing an early start of training. Our patients reported that visualization of muscle activity before actual movements were possible helped them to stay motivated during rehabilitation. Additionally, the visualization of muscle activity increases awareness of the target muscle and facilitates a patient’s understanding as to which motor command leads to the muscle activation.

As is in line with earlier studies (Tung et al., 2003; Bertelli and Ghizoni, 2011; Ray et al., 2011), patients in group 1 attained useful shoulder and upper arm function. In all five patients elbow function improved to a clinically relevant extent with active elbow flexion against gravity (M3) at follow-up. In two patients (Cases 2 and 3) where an Oberlin’s ulnar nerve transfer had been performed, a score of M5 was obtained for elbow flexion. These results are better than those described by Bertelli and Ghizoni (2004) who used the same nerve transfer and obtained scores of M3 to M4. In a retrospective study by Ray et al. (2011) half of 29 patients obtained M4, eight scored M5, while the others had M3 or less, which is comparable to our results. Therefore, the results that were obtained after nerve transfer surgery were similar and in two cases slightly better than those reported in literature. While we believe that a structured rehabilitation protocol using sEMG biofeedback increases patient motivation and awareness, based on our current data we cannot conclude that clinical outcomes can be improved due to the small sample size and the fact that there was no control group. Additionally, it is well known that many factors influence the outcome of peripheral nerve surgery, such as patients’ age and motivation (Novak and Von Der Heyde, 2013), the quality and concept of nerve reconstruction, type of lesion (Moran et al., 2005), etc. Therefore, also in future controlled studies it might be difficult to identify if sEMG can improve clinical outcomes.

For patients with global brachial plexopathies, in whom primary nerve reconstruction and secondary reconstructive procedures have failed to improve hand function, the concept of bionic reconstruction has proven successful to restore hand function via technological means (Aszmann et al., 2015). This novel treatment approach includes surgeries to improve the biotechnological interface, the elective amputation of the functionless hand and subsequent fitting with a mechatronic hand (Aszmann et al., 2015). In this patient population, the control of the prosthetic hand relies on the detection of voluntary residual muscle activity through EMG (Bergmeister et al., 2017). As muscle contraction in these patients will not result in biologically valuable function that is visible to the patient, biofeedback is considered an essential component of rehabilitation.

All patients in group 2 reported that they were highly satisfied with their decision to undergo bionic reconstruction and could reliably control their prosthesis after complementation of rehabilitation. The functional benefit could be confirmed by significant improvements in the ARAT (Action Research Arm Test) from 2.83 ± 4.07 to 25.00 ± 10.94 (*p* = 0.028) on a scoring system from 0 to 57. While this shows the great clinical improvement through bionic reconstruction and sEMG biofeedback training, it still needs to be noted that prosthetic reconstruction cannot fully restore human upper extremity function.

All eleven patients had the possibility to use EMG home training tools to further increase training time. Nine of them decided to take this possibility. The two patients who opted out reported that they did most of the home training protocol (muscle strengthening exercises) outside their home environment and therefore did not like to use an external device. Additionally, they felt that weekly training sessions with the therapist sufficed to improve motor function. Patients who did sEMG home training reported that using the MyoBoy (a simple, two channel EMG device, see Figure 1) made them feel more competent in controlling their EMG signals. As devices for sEMG visualization can be cheap and handy and were described as easy to operate, we strongly recommend their application to supplement therapy in the clinical environment.

Here, we introduced two structured rehabilitation protocols of sEMG-guided training in patients with nerve injuries. While clinical feasibility was proven in eleven patients undergoing structured rehabilitation, further research should include a controlled trial with a larger sample size to estimate the effect of the rehabilitation protocol on functional and psychosocial outcomes.

## Conclusion

Successful neuromuscular rehabilitation requires detailed afferent feedback. Especially in the face of limb loss and/or BP lesions sEMG biofeedback may be used to bridge the time of recovery, where muscle contraction is otherwise unnoticed and help in the cognitively demanding process of establishing new motor patterns that eventually control the prosthetic replacement. After standard nerve transfer surgery individually tailored sEMG biofeedback can facilitate early sensorimotor re-education, enhance patient motivation and compliance and thus improve clinical outcomes.

## Author Contributions

AS, LH, and OA conceived and designed the study. AS and LH performed the data acquisition. LH, AS, and CP analyzed the data. AS, LH, CP, JM, and OA interpreted the data, and wrote and edited the manuscript. All authors gave final approval for publication.

## Conflict of Interest Statement

The authors declare that the research was conducted in the absence of any commercial or financial relationships that could be construed as a potential conflict of interest.

## Abbreviations

BP: brachial plexus
EMG: electromyography
sEMG: surface electromyography
